# Baseline 25-hydroxyvitamin D levels predict the effects of vitamin D supplementation on joint symptoms and cartilage loss in patients with knee osteoarthritis

**DOI:** 10.1093/rap/rkaf061

**Published:** 2025-05-27

**Authors:** Tian Wang, Shuang Zheng, Xingzhong Jin, Han Cen, Zhaohua Zhu, Weiyu Han, Anita Wluka, Flavia Cicuttini, Peihua Cao, Changhai Ding

**Affiliations:** Menzies Institute for Medical Research, University of Tasmania, Hobart, Tasmania, Australia; Division of Rheumatology and Clinical Immunology, Department of Internal Medicine, Beijing United Family Hospital, Beijing, China; Department of Rheumatology, First Affiliated Hospital of Anhui Medical University, Hefei, Anhui, China; Centre for Big Data Research in Health, University of New South Wales, Kensington, New South Wales, Australia; Clinical Research Center, Zhujiang Hospital of Southern Medical University, Guangzhou, Guangdong, China; Clinical Research Center, Zhujiang Hospital of Southern Medical University, Guangzhou, Guangdong, China; Clinical Research Center, Zhujiang Hospital of Southern Medical University, Guangzhou, Guangdong, China; Menzies Institute for Medical Research, University of Tasmania, Hobart, Tasmania, Australia; Menzies Institute for Medical Research, University of Tasmania, Hobart, Tasmania, Australia; Menzies Institute for Medical Research, University of Tasmania, Hobart, Tasmania, Australia; Clinical Research Center, Zhujiang Hospital of Southern Medical University, Guangzhou, Guangdong, China; Menzies Institute for Medical Research, University of Tasmania, Hobart, Tasmania, Australia

**Keywords:** osteoarthritis, knee, vitamin D, 25(OH)D, supplementation, cut-off value, symptomatic, rheumatology, orthopaedics

## Abstract

**Objectives:**

To examine whether the baseline vitamin D level modifies the effects of vitamin D supplementation on knee symptoms and cartilage loss in patients with symptomatic knee OA.

**Methods:**

This was a post hoc analysis for the VIDEO study, which was a large (*n* = 413), randomized, double-blind, placebo-controlled clinical trial in knee OA patients with 25-hydroxyvitamin D [25(OH)D levels ranging from 12.5 to 60 nmol/l]. Knee pain was assessed using the WOMAC pain scale. MRI scans of the knee were obtained. Cartilage volume, cartilage defects (0–4) and bone marrow lesions were measured or graded. Classification trees were applied to categorize the subgroups.

**Results:**

A total of 413 participants (mean age 63.2 years; 50% women) were randomly assigned to vitamin D and placebo groups. A baseline 25(OH)D level of 43 nmol/l was found as the cut-off value. For the total WOMAC score, vitamin D supplementation decreased more than placebo in patients with 25(OH)D levels of 12.5–43 nmol/l (−256.41 *vs* −72.10, *P* = 0.0060) over 2 years but not in those with 25(OH)D levels of 43–60 nmol/l. Comparatively, vitamin D supplementation reduced the total cartilage volume loss (−0.21 *vs* −0.31, *P* = 0.0415) and total cartilage defects progression (0.26 *vs* 0.92, *P* = 0.0029) in patients with 25(OH)D levels of 43–60 nmol/l but not in those with 25(OH)D of 12.5–43 nmol/l.

**Conclusion:**

Supplementation of vitamin D in patients with OA who have 25(OH)D levels ≤43 nmol/l could relieve pain and improve physical function, while in OA patients with 25(OH)D levels >43 nmol/l, supplementation may ameliorate cartilage lesions.

**Trial registration:**

clinicaltrials.gov, NCT01176344; anzctr.org.au, ACTRN12610000495022

## Graphical abstract

**Figure:**
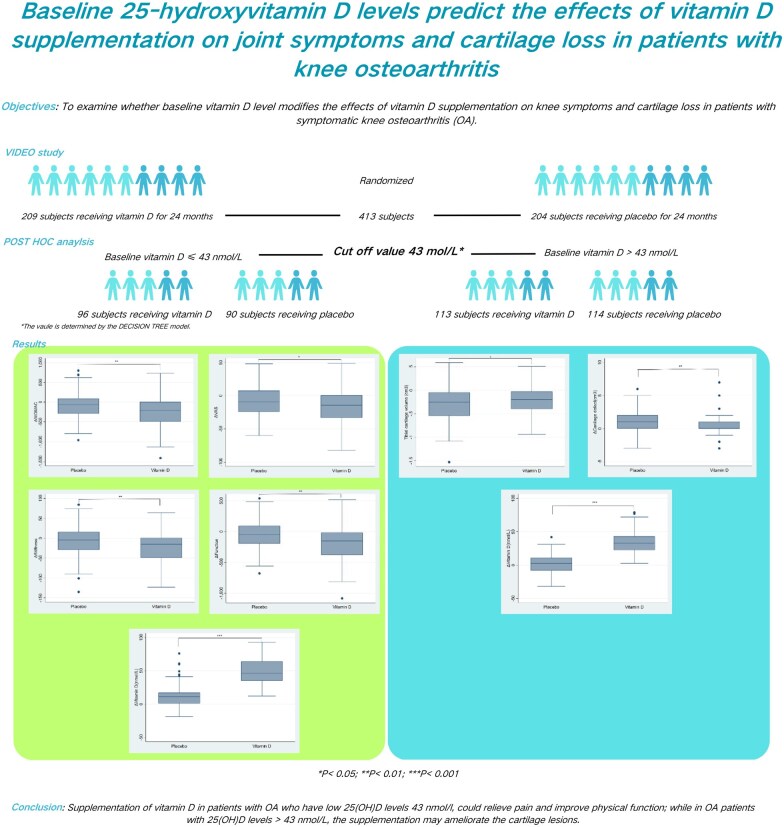

Key messages

- The effects of vitamin D supplementation in patients with OA depend on the baseline level of 25(OH)D, where the cut-off value is 43 nmol/l.
- Supplementation of vitamin D in patients with OA with 25(OH)D levels <43 nmol/l could alleviate pain and improve physical function.
- Supplementation of vitamin D in patients with OA with 25(OH)D levels >43 nmol/l may slow the growth of the cartilage lesions.

## Introduction

OA is a chronic arthritis characterized by joint pain and structural damage that involves not only weight-bearing joints such as knees, hips and ankles [1], but also non-weight-bearing joints such as the hands [2]. The traditional view is that OA is a non-inflammatory joint disease. However, in recent years, evidence has shown that OA is a low-level inflammatory arthritis with an imbalance between osteoprotegerin (OPG) and receptor activator of nuclear factor κB ligand (RANKL) [3–6].

Vitamin D is an endocrine hormone that primarily regulates calcium absorption. It can improve OPG/RANKL imbalance [7]. Clinically, it is used to treat osteoporosis. Recently, evidence has shown that vitamin D has the function of regulating inflammation [8]. Some studies have shown that the application of vitamin D helps to improve SLE and other autoimmune diseases [9]. Whether supplementation with vitamin D can be used to treat OA is an interesting question. Our study on the effects of vitamin D on osteoarthritis (VIDEO) aimed to answer this question. The results showed that vitamin D supplementation did not significantly improve knee pain or cartilage volume loss in patients with knee OA [10]. However, our post hoc analyses showed that vitamin D supplementation had significant effects on knee function [10], effusion synovitis [11], depressive symptoms [12] and foot pain [13], compared with placebo.

The reasons underlying the negative findings of vitamin D supplementation on the primary outcomes in the VIDEO study are unclear. Vitamin D was expected to have positive effects on arthritis, as one cellular experiment revealed that vitamin D can exert a long-lasting anti-inflammatory and anti-proliferative effect on synoviocytes cultured from patients with RA or OA [14]. However, we speculate that outcomes that benefit from vitamin D supplementation may differ among patients with different baseline vitamin D levels. The objective of this study was to examine whether the baseline vitamin D level modifies the effects of vitamin D supplementation on knee symptoms and knee cartilage loss in patients with symptomatic knee OA.

## Methods

### Study design and participants

This was a post hoc analysis of the VIDEO study to identify the distinct responding population for vitamin D supplementation among knee OA patients. The VIDEO study was a large-scale (*n* = 413), randomized, double-blind, placebo-controlled clinical trial in knee OA patients with 25-hydroxyvitamin D [25(OH)D] levels of 12.5–60 nmol/l [10]. It was approved by the Tasmania Health and Human Medical Research Ethics Committee (reference no. H1040) and the Monash University Human Research Ethics Committee (reference no. CF10/1182-2010000616). A detailed description of study methods has been published elsewhere [10] but is briefly summarized here.

Participants, ages 50–79 years, were assigned to either the vitamin D or placebo group at a ratio of 1:1 by computer-generated random numbers. All the participants meet the ACR classification criteria for knee OA. Other clinical conditions that may cause arthralgia or bone pain, such as osteoporosis, autoimmune rheumatic diseases, trauma or surgery, severe viscera lesions and cancer, were excluded. Patients with grade 3 radiographic changes (according to Altman’s atlas) and severe knee pain on standing [>80 mm on 100-mm visual analogue scale (VAS)] were also excluded.

From June 2010 to December 2011, eligible participants with symptomatic knee OA[15] lasting at least 6 months and experiencing pain with a score >20 on a 100-mm VAS and having low levels of 25(OH)D were enrolled in Tasmania and Victoria, Australia and were randomly assigned to treatment with vitamin D3 (cholecalciferol) at 50 000 IU/month (1.25 mg) or placebo for 24 months.

### Assessment of pain

Knee pain was assessed at baseline and at months 3, 6, 12 and 24. Five items of the WOMAC pain scale in the format of a VAS were used to assess pain. The items were summed to create a total pain score (range 0–500). The WOMAC pain score was considered invalid if more than one item was missing. If one item was missing, the average of the remaining four items was calculated and multiplied by 5 to obtain an estimated total pain score. Knee pain on most days of the previous month was also assessed using a VAS (0–100). The total WOMAC score represents the sum of subscale scores including pain, stiffness and physical function.

### MRI assessment of knee structural changes

MRI scans of the study knee were obtained according to a standardized protocol using a 1.5 T whole-body MRI unit with a commercial transmit–receive extremity coil (Picker, Cleveland, OH, USA). The sequences used for cartilage volume assessment were sagittal fat saturated (FS) T1-weighted spoiled gradient echo and cartilage defects and bone marrow lesions were assessed using T2-weighted/proton density–weighted fast spin echo sequences. MRIs were assessed by trained readers blinded to treatments. Cartilage volume was determined using previously described image processing techniques [16]. The volumes of individual cartilage plates (medial tibial and lateral tibial) were isolated by manually drawing the outlines and then resampled by means of bilinear and cubic interpolation for final 3-dimensional rendering using OsiriX Lite imaging software (32-bit version 5.9; Pixmeo SARL, Geneva, Switzerland). The coefficient of variation was 2.1% for the medial tibia and 2.2% for the lateral tibia. Cartilage defects (0–4) were graded on T2-weighted images using a modified Outerbridge classification at the medial tibial, medial femoral, lateral tibial and lateral femoral sites. The presence of cartilage defects was defined as having a score ≥2 at any site. A total score was calculated as the total of subregional scores. Bone marrow lesions, defined as discrete areas of increased signal adjacent to the subcortical bone, were measured using a modified whole-organ MRI score (0, none; 1, ≤25% of the subregion; 2, 25–50%; 3, ≥50%). A total score for the tibiofemoral compartment was calculated as the total of 13 subregional scores (0–39).

### 25(OH)D assays

Serum 25(OH)D was assayed at screening, month 3 and month 24 using direct competitive chemiluminescent immunoassays (DiaSorin, Stillwater, MN, USA).

#### Statistical analysis

This post hoc analysis examined outcomes of alleviation of knee pain, improvement in knee function and structural changes in patients with divergent 25(OH)D levels at baseline. Statistical models were used to identify the distinct response populations within the participants.

A classification tree was applied to justify the robustness of categorizing the subgroups. Classification and regression tree (CART) analysis is a common modelling technique used to make predictions on a variable (*y*), based on several explanatory variables (*x*1, *x*2, …, *x*p). In essence, classification trees are created by finding a single node that maximizes the accuracy and then splitting the data based on this node; using the split data by finding the second-best node to split the data; or repeating the process until we hit an appropriate stopping point (www.stata.com).

Using CART, we found that the cut-off of vitamin D levels was 43 nmol/l. Thus we categorized participants based on vitamin D levels ≤43 or >43 nmol/l to discover intrinsic differences in response to vitamin D treatment between the two groups of patients.

**Figure 1.**
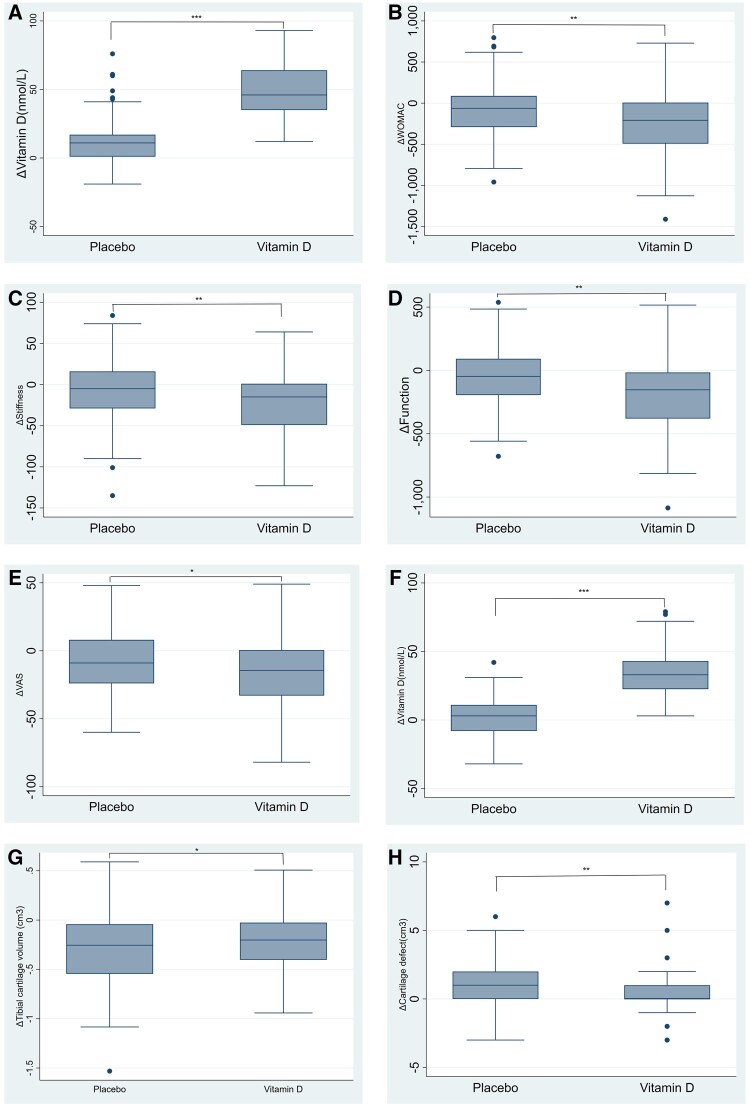
Comparison of changes after intervention between two subgroups. **(A–E)** Changes in the VL group. **(F–H)** Changes in the L group. (A) Comparison of vitamin D changes in the VL group after intervention. (B) Comparison of WOMAC score changes after intervention. (C) Comparison of stiffness changes after intervention. (D) Comparison of function changes after intervention. (E) Comparison of VAS changes after intervention. (F) Comparison of vitamin D changes in the L group after intervention. (G) Comparison of tibial cartilage volume changes after intervention. (H) Comparison of cartilage defect changes after intervention. VL group: participants whose initial vitamin D level was ≤43 nmol/L; L group: participants whose initial vitamin D level was >43 nmol/l

Baseline characteristics were compared between vitamin D groups. Data were analysed using a mixed model with terms for age, sex, BMI, treatment, month and trial centre. An increase in cartilage defects and bone marrow lesions was defined as a change of ≥1 unit in score. To address the correlation, within the repeated measures was addressed by using an individual participant identification as a random effect. The effect of treatment was evaluated by the month ×treatment interaction. Linear regression models with restricted cubic spline terms for baseline vitamin D level were used for graphical displays of the effects of the vitamin D supplementation in relation to categories of baseline vitamin D level (Fig. 1). Multiple imputation using chained equations was used to address missing data caused by loss to follow-up and non-responses. Imputation was performed separately for each treatment group and each outcome using baseline values, age, sex, BMI and serum 25(OH)D level. All statistical analyses were performed using Stata version 15.0 (StataCorp, College Station, TX, USA) and a two-sided *P*-value of 0.05 was considered statistically significant.

## Results

A total of 413 subjects, with a mean age 63.2 years and 50% women, were randomly assigned to two groups: 209 subjects received vitamin D and 204 subjects received placebo [10]. We categorized the participants into two subgroups based on a 25(OH)D level cut-off point of 43 nmol/l [low and very low 25(OH)D groups]. As shown in Table 1, baseline characteristics including total WOMAC score, pain, stiffness, function, VAS score, percentage of cartilage defects and percentage of bone marrow lesions between the vitamin D intervention and placebo groups were largely similar in participants in the low (LI *vs* LP) and very low (VLI *vs* VLP) 25(OH)D groups, respectively. Tibial cartilage volume was also not significantly different between the two groups in participants with very low 25(OH)D. However, there were significant differences in the tibial cartilage volume between the intervention and placebo groups (*P* < 0.05) in participants with low (LI *vs* LP) 25(OH)D.

**Table 1.** Baseline characteristics of participants classified by vitamin D levels at baseline

Characteristics	LI group (*n* = 113)	LP group (*n* = 114)	*P* value	VLI group (*n* = 96)	VLP group (*n* = 90)	*P*-value
Age, years, mean (s.d.)	64.06 (7.41)	62.31 (7.13)	0.07	62.94 (6.17)	63.54 (7.30)	0.54
Women, *n* (%)	60 (53.10)	49 (42.98)	0.13	46 (47.92)	53 (58.89)	0.14
BMI (s.d.)	29.38 (5.11)	29.26 (4.10)	0.84	29.79 (5.71)	30.12 (5.18)	0.68
Serum 25(OH)D, nmol/l, mean (s.d.)	52.77 (5.59)	53.30 (5.41)	0.47	33.10 (7.52)	31.80 (8.20)	0.26
Radiographic OA, *n* (%)	95 (94.06)	89 (96.74)	0.45	94 (97.92)	87 (96.67)	0.42
Total WOMAC score (0–2400), mean (s.d.)	798.85 (441.16)	584.78 (357.24)	0.21	892.00 (506.34)	752.14 (414.91)	0.52
Pain (0–500), mean (s.d.)	173.07 (85.73)	127.10 (76.59)	0.18	174.33 (109.87)	145.30 (74.64)	0.51
Stiffness (0–200), mean (s.d.)	69.4 (39.93)	68.80 (31.71)	0.97	73.22 (30.74)	75.50 (45.27)	0.91
Function (0–1700), mean (s.d.)	556.38 (350.15)	388.88 (267.51)	0.21	644.45 (380.23)	531.34 (323.82)	0.49
VAS pain (0–100), mean (s.d.)	51.35 (17.34)	50.33 (18.19)	0.67	51.45 (20.41)	48.60 (17.28)	0.31
Tibial cartilage volume, cm^3^, mean (s.d.)	3.42 (1.02)	3.82 (1.04)	**0.004**	3.52 (1.06)	3.41 (0.99)	0.47
Cartilage defects, *n* (%)	90 (81.08)	93 (81.58)	0.11	75 (78.95)	72 (80.90)	0.65
Bone marrow lesions, *n* (%)	90 (81.08)	94 (82.45)	0.11	73 (76.84)	68 (72.40)	0.47

Significant values in bold.

VLI: vitamin D intervention with very low baseline vitamin D (≤43 nmol/l); VLP: placebo intervention with very low baseline vitamin D (≤43 nmol/l); LI: vitamin D intervention with low baseline vitamin D (>43–<60 nmol/l); LP: placebo intervention with low baseline vitamin D (>43–<60 nmol/l).

There was no statistical difference in baseline 25(OH)D levels between the intervention and placebo subgroups (VLI *vs* VLP, mean 33.10 nmol/l *vs* 31.80 nmol/l, *P* = 0.7075; LI *vs* LP, mean 52.77 nmol/l *vs* 53.30 nmol/l, *P* = 0.5059). However, the differences in the 25(OH)D levels after 24 months (VLI *vs* VLP, mean 82.09 nmol/l *vs* 44.73 nmol/l, *P* < 0.0001; LI *vs* LP, mean 85.91 nmol/l *vs* 55.05 nmol/l, *P* < 0.0001) and the change in the 25(OH)D level over 24 months (VLI *vs* VLP, mean 49.62 nmol/l *vs* 12.25 nmol/l, *P* < 0.0001; LI *vs* LP, mean 32.87 nmol/l *vs* 2.02 nmol/l, *P* < 0.0001) between groups were statistically significant. Collectively, the 25(OH)D levels improved in both the vitamin D and placebo groups, but the increase was more greater in vitamin D intervention groups (VLI and LI) (Tables 2 and 3).

**Table 2.** Comparisons of changes in joint symptoms and structures with baseline 25(OH)D levels ≤43 nmol/l (VL group).

Characteristics	Vitamin D (*n* = 96)			Placebo (n = 90)			Between-group difference in change, mean (95% CI)	*P*-value
	Mean (95% CI)		Change, mean (95% CI)	Mean (95% CI)		Change, mean (95% CI)		
	Baseline	Month 24		Baseline	Month 24			
Vitamin D, nmol/l	33.10 (31.58, 34.63	82.09 (77.52, 86.66)	49.62 (45.05, 54.19)	31.80 (30.08, 33.52)	44.73 (40.70, 48.76)	12.25 (8.22, 16.28)	−37.37 (−43.31, −31.43)	**<0.0001**
Total WOMAC score (0–2400)	892.00 (265.52, 1462.16	400.27 (297.99, 502.56)	−256.41 (−345.37, −167.45)	752.14 (478.69, 892.79)	549.29 (456.92, 641.65)	−72.10 (−162.26, 18.07)	184.31 (52.97, 315.65)	**0.0060**
Pain (0–500)	174.33 (22.19, 302.55)	85.85 (65.24, 106.47)	−47.75 (−75.38, −20.12)	145.30 (123.23, 184.28)	102.55 (83.49, 121.60)	−27.53 (−50.53, −4.53)	20.22 (−15.82, 56.25)	0.2701
Stiffness (0–200)	73.22 (38.67, 102.44)	36.89 (27.71, 46.08)	−25.67 (−36.48, −14.85)	75.50 (40.65, 98.90)	51.03 (42.43, 59.62)	−6.97 (−16.00, 2.06)	18.70 (4.58, 32.81)	**0.0096**
Function (0–1700)	644.45 (193.02, 1068.37)	281.13 (207.25, 355.00)	−187.82 (−250.77, −124.87)	531.34 (293.49, 631.93)	396.00 (328.78, 463.23)	−38.54 (−102.44, 25.36)	149.28 (56.29, 242.28)	**0.0017**
VAS pain (0–100)	51.45 (47.64, 55.21)	31.11 (25.01, 37.21)	−15.82 (−22.13, −9.51)	48.60 (44.71, 52.50)	37.31 (31.65, 42.97)	−6.87 (−12.60, −1.14)	8.95 (0.22, 17.68)	**0.0445**
Tibial cartilage volume, cm^3^	3.52 (3.29, 3.62)	3.21 (3.02, 3.41)	−0.25 (−0.34, −0.17)	3.41 (3.32, 3.65)	3.24 (3.06, 3.42)	−0.28 (−0.36, −0.21)	−0.03 (−0.14, 0.09)	0.6357
Cartilage defect	14.54 (13.75, 15.34)	15.64 (14.71, 16.57)	0.58 (0.17, 1.00)	14.17 (13.35, 15.00)	14.35 (13.46, 15.23)	0.32 (−0.04, 0.69)	−0.26 (−0.81, 0.28)	0.3477
Bone marrow lesions	2.73 (2.10, 3.36)	2.56 (1.82, 3.29)	−0.20 (−0.78, 0.37)	3.35 (2.70, 4.01)	2.98 (2.21, 3.76)	−0.05 (−0.59, 0.48)	0.15 (−0.66, 0.95)	0.7170

Significant values in bold.

Changes in outcomes are generated from mixed effects models adjusted for age, sex and BMI.

Between-group differences were calculated by subtracting the vitamin D group values from the placebo group values.

**Table 3.** Comparisons of changes in joint symptoms and structures with baseline 25(OH)D levels >43 nmol/l (L group).

	Vitamin D (*n* = 113)			Placebo (*n* = 114)			Between-group difference in change, mean (95% CI)	*P*-value
	Mean (95% CI)			Mean (95% CI)		Change, mean (95% CI)		
Characteristics	Baseline	Month 24	Change, mean (95% CI)	Baseline	Month24			
Vitamin D, nmol/l	52.77 (51.73, 53.81)	85.91 (82.76, 89.06)	32.87 (29.73, 36.02)	53.30 (52.29, 54.30)	55.05 (51.62, 58.49)	2.02 (−1.42, 5.46)	−30.85 (−35.52, −26.19)	**<0.0001**
Total WOMAC score (0–2400)	798.85 (576.02, 997.31)	447.29 (361.90, 532.69)	−230.39 (−316.60, −144.17)	584.78 (308.74, 694.69)	480.28 (382.80, 577.75)	−212.47 (−307.38, −117.55)	17.92 (−113.23, 149.06)	0.7882
Pain (0–500)	173.07 (129.69, 213.21)	83.82 (66.67, 100.97)	−57.30 (−76.74, −37.85)	127.10 (70.90, 159.75)	96.02 (77.66, 114.39)	−45.62 (−66.26, −24.98)	11.68 (−16.43, 39.79)	0.4152
Stiffness (0–200)	69.40 (51.92, 82.47)	43.02 (34.29, 51.76)	−16.63 (−25.98, −7.29)	68.80 (51.99, 71.07)	42.81 (33.64, 51.98)	−25.98 (−35.72, −16.24)	−9.34 (−22.72, 4.03)	0.1709
Function (0–1700)	556.38 (370.72, 723.89)	316.98 (254.44, 379.51)	−159.39 (−221.37, −97.41)	388.88 (178.53, 468.85)	344.74 (272.89, 416.58)	−140.42 (−208.66, −72.17)	18.97 (−75.32, 113.26)	0.6924
VAS pain (0–100)	51.35 (47.84, 54.46)	35.90 (30.68, 41.13)	−14.60 (-20.71, -8.49)	50.33 (47.29, 53.94)	35.34 (29.55, 41.13)	−11.36 (-18.08, -4.65)	3.24 (-5.97, 12.45)	0.4898
Tibial cartilage volume, cm^3^	3.42 (3.34, 3.64)	3.30 (3.14, 3.46)	−0.21 (−0.28, −0.14)	3.82 (3.59, 3.89)	3.43 (3.26, 3.61)	−0.31 (−0.38, −0.24)	−0.10 (−0.20, −0.0040)	**0.0415**
Cartilage defect	15.04 (14.33, 15.75)	15.28 (14.53, 16.03	0.26 (−0.03, 0.55)	14.69 (13.98, 15.39)	15.72 (14.93, 16.51)	0.92 (0.60, 1.24)	0.66 (0.23, 1.10)	**0.0029**
Bone marrow lesions	3.50 (2.88, 4.12)	3.43 (2.72, 4.15)	−0.10 (−0.62, 0.42)	3.79 (3.18, 4.40)	4.18 (3.42, 4.93)	0.38 (−0.23, 0.98)	0.48 (−0.32, 1.28)	0.2420

Significant values in bold.

Changes in outcomes are generated from mixed effects models adjusted for age, sex and BMI.

Between-group differences were calculated by subtracting the vitamin D group values from the placebo group values.

For the total WOMAC score, vitamin D supplementation decreased more than placebo in participants with very low 25(OH)D levels (VLI *vs* VLP: −256.41 *vs* −72.10, *P* = 0.0060) over 2 years. Similar trends were seen in WOMAC stiffness and WOMAC function, as well as VAS pain (Table 2). In contrast, vitamin D supplementation did not decrease the total WOMAC score (LI *vs* LP: −230.39 *vs* −212.47, *P* = 0.7882) as well as other symptomatic outcomes in participants with low 25(OH)D levels (Table 3). In addition, after 24 months, although vitamin D supplementation could reduce WOMAC pain in both the vitamin D low and very low groups, the differences between the treatment group and placebo group were not statistically significant (Tables 2 and 3).

For knee structural changes (Tables 2 and 3), vitamin D supplementation, compared with placebo, did not significantly reduce cartilage volume loss and cartilage defects progression in participants with very low 25(OH)D levels, but these effects were significant in those with low 25(OH)D levels (LI *vs* LP: −0.21 *vs* −0.31, *P* = 0.0415 for total tibial cartilage volume loss; and 0.26 *vs* 0.92, *P* = 0.0029 for total cartilage defects progression). There were no significant effects of vitamin D supplementation on bone marrow lesions in participants with both low and very low 25(OH)D levels.

## Discussion

This study analysed the effects of vitamin D supplementation on knee symptoms and structural changes in patients with knee OA based on baseline vitamin D levels. We found that vitamin D supplementation had effects on knee symptoms in patients with very low 25(OH)D levels (≤43 nmol/l) and it hindered cartilage lesions in those with low 25(OH)D levels (>43 nmol/l–<60 nmol/l).

Vitamin D deficiency is a worldwide pandemic, but there is no consensus to define the disorder. The European Society for Clinical and Economic Aspect of Osteoporosis and Osteoarthritis recommends that 50 nmol/l should be the minimal serum 25(OH)D concentration at the population level [17]. The US Centers for Disease Control and Prevention and the International Osteoporosis Foundation believe that 25(OH)D levels should be at least 30 ng/ml (74.88 nmol/l) in healthy adults, while the Mayo Clinic thinks the optimal value of 25(OH)D is between 25 ng/ml (62.4 nmol/l) and 80 ng/ml (199.68 nmol/l) [18]. The severity of vitamin D deficiency is divided into mild, moderate and severe. Mild deficiency is defined as a 25(OH)D level of 10– 20 ng/ml (25–50 nmol/l), moderate deficiency as 5–10 ng/ml (12.5–25 nmol/l) and severe deficiency as <5 ng/ml (12.5 nmol/l) [19]. Some studies regarded a serum vitamin D level of 25 nmol/l as the cut-off value for a ‘low’ vitamin D level. In the VIDEO study, the baseline vitamin D level of most participants was of moderate or mild deficiency.

Although the VIDEO study showed that neither symptom remission nor cartilage protection occurred with vitamin D supplementation in symptomatic knee OA patients with low serum 25(OH)D levels in the analyses of the primary endpoints, some symptomatic indicators were improved in the post hoc analyses. Thus we believe some participants would benefit from vitamin D intervention. A previous study reported that vitamin D supplementation had significant effects on bone mineral density at the spine and hip only in those with baseline 25(OH) D levels <30 nmol/l [20]. We hypothesized that some patients in the study, especially with very low levels of vitamin D, could benefit from vitamin D supplementation. Thus we divided the participants into subgroups based on the threshold (43 nmol/l), which was generated by CART models. CART (also called decision tree) models are classifiers that predict class labels for data items [21]. The participants could be divided into two subgroups in the intervention (vitamin D supplement) or placebo group and the cut-off value for their initial 25(OH) D level was 43 nmol/l. Some studies, especially clinical trials, used decision tree models to classify their subjects into subgroups or categories [22, 23].

We found that supplementation of vitamin D could benefit symptom alleviation in knee OA in patients with 25(OH)D levels ≤43 nmol/l and for cartilage protection in patients with 25(OH)D levels >43 nmol/l. In the participants with 25(OH)D levels ≤43 nmol/l, the VAS for pain had a greater improvement in VLI subgroup. The total WOMAC score was significantly reduced in the VLI subgroup after 2 years of intervention, but this phenomenon was not seen in the VLP subgroup. In-depth analysis of stiffness and function after 2 years of intervention in the VLI subgroup showed the score changes improved significantly compared with the VLP subgroup. These results indicate that for OA patients with 25(OH)D levels ≤43 nmol/l, supplementation with vitamin D could effectively improve their joint symptoms and function.

One study utilized vitamin D to alleviate chronic pain. Yilmaz *et al.* [24] administered vitamin D (50 000 IU/week oral vitamin D3 for 3 months) to patients with non-specific chronic widespread musculoskeletal pain and vitamin D deficiency [25(OH)D <25 ng/ml]. The vitamin D treatment provided improvements in musculoskeletal symptoms, level of depression and the quality of life of patients [24]. The underlying mechanism for the analgesic effects of vitamin D are unclear, but it may be related to its anti-inflammatory effects. Huhtakangas *et al.* [14] reported that both 1,25-dihydroxvitamin D3 [1,25(OH)_2_D_3_] and calcipotriol inhibited synovial stromal cell (SSC) proliferation for up to 23 days. 1,25(OH)_2_D_3_ and calcipotriol reduced the secretion of most inflammatory factors. In addition, the level of IL-6 was still diminished at 10 days after exposure, emphasizing the long-term impact of calcipotriol on SSCs. This research illustrated the potential role of vitamin D in symptomatic OA by inhibition of production of inflammatory cytokines.

We found that in the participants with vitamin D >43 nmol/l, there was no difference between the intervention group and placebo group in either the VAS pain or total WOMAC pain scores. However, for the change in the tibial cartilage volume and the cartilage defect, vitamin D supplementation had better effects than placebo treatment.

Kuyucu *et al.* [25] applied intra-articular injection of 1,25(OH)_2_D_3_ to rats with cartilage defects and confirmed local use of 1,25(OH)_2_D_3_ was therapeutic for joint cartilage damage. By observation of ovariectomized rats, Li *et al.* [26] reported that cartilage erosion due to ovariectomy was significantly ameliorated in a dose-dependent manner by 1,25(OH)_2_D_3_ supplementation but was exacerbated by a vitamin D–deficient diet (VDD). In histological experiments, the expression of TGF-β1 and type II collagen in articular cartilage was suppressed by ovariectomy and VDD and rescued by 1,25(OH)_2_D_3_ supplementation. The expression of MMP-9 and MMP-13 in articular cartilage increased with ovariectomy and VDD and decreased after supplementation with 1,25(OH)_2_D_3_. Tetlow *et al.* [27] found that vitamin D receptors (VDRs) were expressed in chondrocytes from osteoarthritic cartilage rather than from normal cartilage. VDR expression in osteoarthritic cartilage was often associated with sites where MMP expression was prevalent, an observation that contrasts with their virtual absence in normal age-matched cartilage. Furthermore, increased production of MMP-9 and prostaglandin E2 stimulated with phorbol myristate acetate in osteoarthritic cartilage was significantly suppressed by treatment with 1,25(OH)_2_D_3_. The results of this study imply that osteoarthritic chondrocytes up-regulate the expression of VDRs and increased their sensitivity to 1,25(OH)_2_D_3_ and thus 1,25(OH)_2_D_3_ might protect cartilage by inhibition of MMP.

Vitamin D supplementation had effects on symptoms but did not have effects on cartilage in those with 25(OH)D levels ≤43 nmol/l, while 25(OH)D levels >43 nmol/l had effects on cartilage but did not have effects on symptoms. We reported that moderate vitamin D deficiency [25(OH)D levels <25 nmol/l] predicted incident or worsening knee pain over 5 years, indicating that correcting moderate vitamin D deficiency may attenuate worsening of knee pain in elderly people but giving supplements to those with higher 25(OH)D levels is unlikely to be effective [28]. This association was independent of knee structural changes such as osteophytes, joint space narrowing and cartilage defects [28]. We speculate that vitamin D could modify neuropathic pain, which is increased in those with moderate vitamin D deficiency [29]. In contrast, vitamin D signalling on cartilage may be impaired in those with moderate to severe vitamin D deficiency [30] and thus vitamin D supplementation was only effective on cartilage for those with mild vitamin D deficiency.

There were several limitations in this study. First, it was a post hoc analysis of the VIDEO study, which was not the originally design. The participants did not include patients whose vitamin D levels were <12.5 nmol/l (5 ng/ml). These patients may also benefit from supplementation of vitamin D based on our results. Second, in this post hoc study, it cannot be determined whether sunlight exposure could affect our observed associations, as we did not collect these data. Third, we did not verify the mechanisms of how vitamin D supplementation has a beneficial effect on either joint symptoms or joint structures. Experimental studies are needed to explore this in the future. Last, a follow-up period of 2 years may not be long enough to observe changes in cartilage measures. Further trials are needed to explore whether vitamin D has long-term effects on joint structures.

In conclusion, supplementation of vitamin D could relieve pain and improve physical function in knee OA patients with 25(OH)D levels ≤43 nmol/l and slow the growth of cartilage lesions in those with 25(OH)D levels >43 nmol/l.

## Data Availability

Data are available in the article.
